# Suboptimal extracellular pH values alter DNA damage response to induced double‐strand breaks

**DOI:** 10.1002/2211-5463.12384

**Published:** 2018-02-16

**Authors:** Julien Massonneau, Camille Ouellet, Fabrice Lucien, Claire M. Dubois, Jessica Tyler, Guylain Boissonneault

**Affiliations:** ^1^ Department of Biochemistry Faculty of Medicine & Health Sciences Université de Sherbrooke Quebec Canada; ^2^ Department of Pediatry Faculty of Medicine & Health Sciences Université de Sherbrooke Quebec Canada; ^3^ Department of Pathology and Laboratory Medicine Weill Cornell Medical College New York NY USA

**Keywords:** DNA double‐stranded breaks, DNA repair, genetic instability, histones, pH

## Abstract

Conditions leading to unrepaired DNA double‐stranded breaks are potent inducers of genetic instability. Systemic conditions may lead to fluctuation of hydrogen ions in the cellular microenvironment, and we show that small variations in extracellular pH, termed suboptimal pHe, can decrease the efficiency of DNA repair in the absence of intracellular pH variation. Recovery from bleomycin‐induced DNA double‐stranded breaks in fibroblasts proceeded less efficiently at suboptimal pHe values ranging from 7.2 to 6.9, as shown by the persistence of repair foci, reduction of H4K16 acetylation, and chromosomal instability, while senescence or apoptosis remained undetected. By allowing escape from these protective mechanisms, suboptimal pHe may therefore enhance the genotoxicity of double‐stranded breaks, leading to genetic instability.

**Abbreviations**
CBMN: Cytokinesis‐block micronucleus
DDR: DNA damage response
DSBs: Double‐stranded breaks
pHe: Extracellular pH
pHi: Intracellular pH
ROS: Reactive oxygen species
SSB: Single‐stranded break
TdT: Terminal deoxynucleotidyl transferase

Conditions leading to stabilization of DNA double‐stranded breaks (DSBs) may be potent inducers of genetic instability 1. Unrepaired or misrepaired DSBs can result in senescence or induced apoptosis, but can also lead to mutagenesis and chromosomal aberrations. The latter includes translocations and deletions that can result in loss of heterozygosity and carcinogenesis. DSBs arise in cells under the influence of external factors, such as oxidative stress and ionizing radiation, but they are also associated with physiological processes such as DNA replication. Local variations in pH may be considered such as ‘external factor’ when it arises as a result of systemic conditions leading to fluctuation of hydrogen ions in the cellular microenvironment. Experimentally, severe subphysiological decreases in pHe (6.5 or below) have been previously reported to induce clastogenic effects such that chromosomal aberrations were rapidly observed 2, 3. Such a low pHe is, however, more typical of severe hypoxic conditions generated from tumor cell metabolism producing lactate from anaerobic glycolysis 4, 5. These cells create a microenvironment that is well known to increase mutagenicity, metastatic capacity, resistance to chemotherapy, and invasiveness 4, 6, 7. In normal cells however, the genetic consequence of pHe falling slightly below 7.4 has never been reported. These ‘suboptimal pHe’ may often be observed under systemic pathological conditions such as renal failure, diabetes, alcohol excess or starvation leading to transient metabolic acidosis characterized by a nonlethal decrease in blood pH to values down to 7.0 8, 9, 10, 11. However, suboptimal pHe may also be observed locally in tissue microenvironment as a result of ischemia, inflammation, or alteration of cellular metabolism in different organs 12, 13, 14. Whether suboptimal pHe impacts intracellular pH (pHi) is unknown as the latter must be maintained within a narrow range to prevent disruption of basic cell functions including membrane permeability, enzyme activity, and cellular metabolism among others 15. It also remains to be established whether such a moderate decrease in pHe alters DNA repair and genetic integrity.

Bleomycin is a radiomimetic that functions as a catalyst to produce reactive oxygen species (ROS) leading to clusters of oxidized DNA lesions, single‐strand breaks, and DSBs similar to ionizing radiation 16. Cell exposure to this drug leads to formation of DSBs that are normally repaired within minutes once cells are returned to drug‐free culture media 17. By achieving careful pH control of normal fibroblasts' culture media, we show that even a slight departure from the optimal pH value of 7.4 reduces the repair kinetics of bleomycin‐induced DSBs and chromosomal instability. As we further demonstrate that these cells are not committed to either senescence or apoptosis processes, the delayed kinetics of DSB repair at suboptimal pHe may therefore increase genetic instability and genomic rearrangements in replicating cells. This may represent a potent adjuvant mechanism for carcinogenesis especially in those cells harboring inherited genetic instability such as that resulting from mutations in repair genes 18, 19.

## Materials and methods

### Cell culture

Fibroblasts from fetal human skin were purchased from Coriell Institute (GM05388A). Cells were cultured in high‐glucose DMEM commercial media (5 g·L^−1^) supplemented with FBS 10%, glutamate, nonessential amino acids, penicillin, and streptavidin antibiotics. Suboptimal pHe media were freshly prepared from DMEM powder (D5030, Sigma, St. Louis, MO, USA) and supplemented with 1 g·L^−1^ of glucose, glutamine, sodium pyruvate, nonessential amino acids, 10% FBS, penicillin, and streptavidin antibiotics. Sodium bicarbonate was added at a final concentration of 17 mm, 12 mm, 7 mm, 5.7 mm, and 2.4 mm so as to obtain pH values of 7.4, 7.2, 7.0, 6.9, and 6.5, respectively, after fine adjustment with HCl. Cells were harvested by trypsin treatment.

### Bleomycin treatment

Once cells reached 70% confluence, DNA DSBs were induced by addition of bleomycin sulfate to the DMEM, adjusted to pH 7.4, at a final concentration of 100 μg·mL^−1^ (Cayman Chemical, #13877, Ann Arbor , MI, USA). Cells were harvested following 1 h of treatment and washed thrice with PBS. Fresh DMEM at different pH values was added without bleomycin to initiate post‐treatment recovery at suboptimal pHe values under 5% CO_2_.

### Immunofluorescence

For all experiments, cells were plated and fixed on glass coverslips for 5 min using 3.7% w/w formaldehyde and incubated for 30 min in blocking solution (3% BSA, 0.1% Triton X‐100 in 1 × PBS). Cells were incubated with primary antibodies diluted in blocking solution to 1 : 500 for γH2AFX mouse (#613402, BioLegend), to 1 : 1000 rabbit 53BP1 (#NB100‐304, Novus, Oakville, ON, Canada) or 1 : 100 H4K16ac rabbit (#ab109463, Abcam, Cambridge, MA, USA). Cy3‐labeled mouse and Cy5‐labeled rabbit secondary antibody and Cy5‐labeled rabbit diluted 1 : 200 were used for detection (715‐165‐150, Jackson ImmunoRes, West Grove, PA, USA). DAPI was then added with the ProLong Diamond Antifade (P36962, ThermoFisher). Images were taken using a Leica DM2500 Optigrid fluorescence microscope and processed with the ImageJ fiji software.

### Immunoblots

Harvested cells were resuspended in PBS and lysed at 100 °C. Ten micrograms of proteins lysate was resolved by SDS/PAGE followed by transfer onto a nitrocellulose membrane. Incubation in nonfat dry milk (5%) was used as blocking step. γH2AFX, MRE11 (4895, Cell Signaling), and H4K16ac antibodies were both used at 1 : 1000 dilution in blocking solution. DyLight 680 (35518, ThermoScientific, Waltham, MA, USA) and DyLight 800 (DkxRb‐003‐F800NHSX, Jackson ImmunoRes) secondary antibodies were used at a dilution of 1 : 10 000. Fluorescence detection was performed using a LI‐COR Odyssey system.

### Pulsed‐field Gel Electrophoresis (PFGE)

DNA from cells resuspended in 50 μL of PBS was isolated using the Dynabeads SILANE Genomic DNA Kit (37012D, ThermoFisher) according to the manufacturer's protocol. Five hundred nanograms of DNA were loaded onto a 1% agarose gel for PFGE. PFGE in 0.5 × TBE buffer was run for 18 h at 15 °C with 1 to 15 s switch time and 5 volt·cm^−1^. DNA migration analyses were performed with imagequant software.

### Determination of DSBs by qTUNEL

A modification of the terminal deoxynucleotidyl transferase (TdT) nick‐end labeling method has been developed by our group allowing for the relative quantification of DSBs (qTUNEL) 20. Briefly, an initial step of DNA nick and gap repair is performed using a combination of T4 DNA ligase and T4 DNA polymerase prior to radioactive labeling using 0.13 mm of dATP, [α‐^32^P], and 0.013 mm ddATP. In these conditions, complete removal of the DNA nicks and gap within genomic DNA preparation was achieved. As a positive control, digestion of the DNA was performed with 2 μL of fragmentase (M0348, NEB, Ipswich, MA, USA).

### Intracellular pH measurement

Intracellular pH (pHi) was determined by confocal microscopy using the fluorescent pH‐sensitive probe BCECF (2,7‐bis‐(2‐carboxyethyl)‐5‐(and‐6)‐carboxyfluorescein, acetoxy‐methyl ester) (B1170, ThermoFisher) as described in Ref. 21. Fibroblasts were exposed to suboptimal pHe values as given and for 72 h. BCECF was added to the cells' culture media at a final concentration of 2 mm for 20 min at 37 °C. Cells' culture dishes were placed under a confocal microscope (Olympus FV1000) and excited at 450 nm and 488 nm. Emitted fluorescence was collected at 535 nm. Fluorescence ratios were converted to pH values with a calibration curve obtained as described by Tekpli *et al*. 22.

### Cytokinesis‐block micronucleus (CBMN) assay

After bleomycin treatment as described above, cells were incubated 48 h in suboptimal pH. Cytochalasin B (C6762, Sigma) was added in the media 24 h following bleomycin treatment. Cells, plated on glass coverslip, were fixed for 5 min with 3.7% w/w formaldehyde. DAPI was then added with the ProLong Diamond Antifade (P36962, ThermoFisher).

### Determination of senescence

Cells were incubated for 5 days at given suboptimal pHe in 6‐cm well plates and harvested by trypsin treatment. Cells were resuspended in 100 μL of staining solution containing 40 mm citric acid/sodium phosphate pH 6.0, 0.15 m NaCl, 0.2 mm MgCl_2_ supplemented with 1 × protease inhibitor cocktail (B14001, Biotool), and 10 μg·μL^−1^ of ONPG. An equal number of cells were lysed by five freeze–thaw cycles, incubated for 3 h at 37 °C and the O.D. read at 420 nm. H_2_O_2_‐induced senescence was used as a positive control and measured following 4 days of recovery.

### Determination of apoptosis

Immunoblots were performed as described above. Rabbit antibody against cleaved caspase rabbit (sampler kit 9929, Cell Signaling) was used at a dilution of 1 : 1000. Cell treatment with 1 μm of staurosporine (ALX‐380‐014, Enzo Life Sciences) was used as a positive control. Cells were harvested immediately following treatment.

### Cell proliferation assay

Cell viability was performed essentially as described by Feoktistova *et al*. 23. Briefly, thirty thousand cells were plated 24 h before BLM treatment. Cells were allowed to recover and proliferate for 96 h post‐treatment at all given suboptimal pHe. Cells were rinsed with 10 mL of PBS and fixed with 2 mL of acetic acid and methanol solution (1 : 7 vol/vol) for 5 min. Cells were stained with 0.5% crystal violet (B21932, Thermo Scientific) at room temperature for 2 h. Cell dishes were washed with water to rinse off crystal violet. Cells were air‐dried for 2 h, and images were taken using a Leica DM2500 Optigrid microscope. For each condition, proliferation was inferred from determination of cell density/microscope fields (*n* = 5).

## Results and Discussion

**Figure 1:**
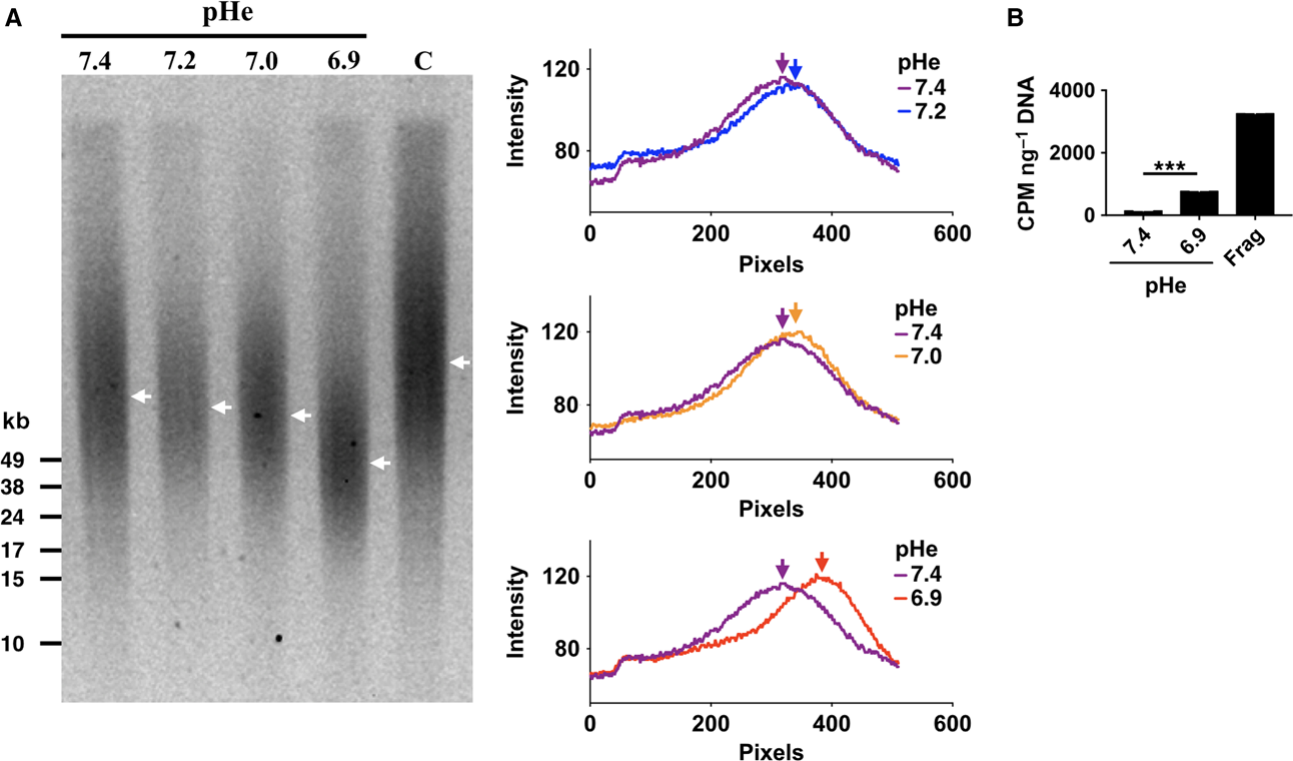
DSBs formation at suboptimal pHe. (A) Left panel: PFGE in neutral conditions to resolve DSBs. Arrows show maximal density (apex) for each track. Right panel: corresponding densitometric scan of the PFGE tracks shown in A. Arrows indicate the corresponding position of apex shown on the gel. (B) qTUNEL quantification of DNA double‐stranded breaks for the highest and lowest values of the suboptimal pHe range. Frag indicates fragmentase‐digested DNA from fibroblasts used as a positive control. Values are mean ± SEM of three determinations.

Normal human embryonic fibroblasts were cultured to 70% confluence and the commercial media supplemented with bleomycin as described in Materials and methods. Cells were allowed to recover for 15 min in the absence of the drug at given suboptimal pHe values (Fig. 1). Pulse‐field gel electrophoresis in neutral condition was then used to assess relative variation in DNA DSBs. As the genomic DNA is obtained from a population of cells, DSBs must be present in a significant fraction of the cells and occur at several loci/cell to observe an increase in DNA mobility. As shown in Fig. 1, suboptimal pHe values of 7.2 and 7.0 induce a detectable increase in DNA mobility relative to the pH 7.4 reference value, whereas an even greater mobility was observed following further reduction of pHe from 7.0 to pH 6.9 (Fig. 1A, right panel). The number of mechanical DNA breaks induced by the extraction procedure is expected to be similar across samples and accounts for the basal DNA mobility that can be observed in the control sample. The relative increase in mobility between samples is therefore expected to result from alterations in the repair of bleomycin‐induced DSBs. DNA 3'OH ends are expected to result from bleomycin action, so we determined the relative extent of 3'OH at DSBs using a modification of the TUNEL assay termed ‘qTUNEL’ allowing specific radioactive detection of DSBs following an initial step of nicks and gaps repair 20. As shown in Fig. 1B, persistence of 3'OH DSBs is confirmed when shifting the pH to 6.9 during the repair process. As expected, a much sharper increase in DSBs is generated from fragmentase digestion used as a positive control for enzyme‐induced breaks. Results from the qTUNEL assay are therefore in accordance with the PFGE and confirm that bleomycin‐induced DSBs are processed with a slower kinetics at suboptimal pHe.

**Figure 2:**
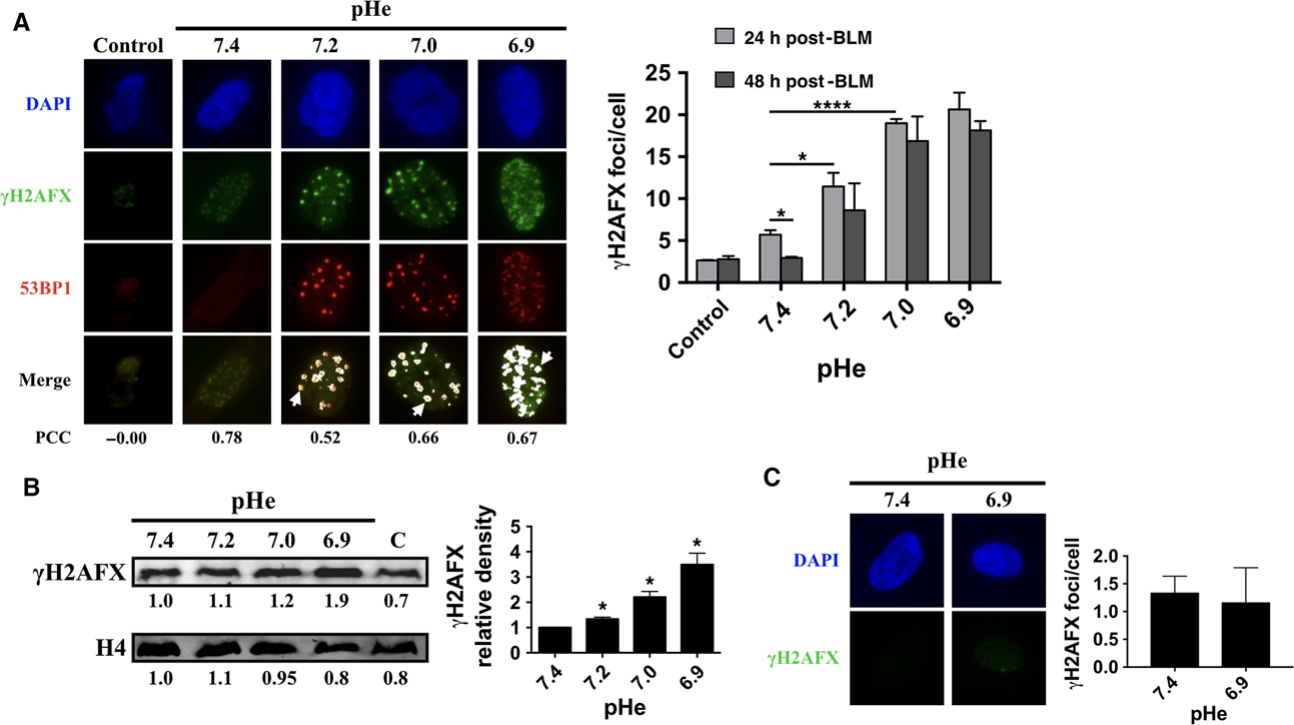
Persistence of repair foci at suboptimal pHe. (A) Left panel shows immunofluorescence detection of γH2AFX and 53BP1 following 24 h recovery from bleomycin treatment. Control, sample before damage induction. Colocalization is shown in white; PCC, Pearson correlation coefficient score. Right panel corresponding to γH2AFX foci/cell at 24 h and 48 h following recovery. Only foci larger than 10 pixels were counted. Experiments were performed in triplicate. Values are mean ± SEM. *P < 0.05, ****P < 0.00005. (B) Immunoblots for the detection of γH2AFX and histone H4. Right panel shows γH2AFX/H4 ratios at indicated pHe values. C, control before damage induction. Quantitation of three independent immunoblots is shown on the right. Values are mean ± SEM. *P < 0.05. (C) Immunofluorescence detection of γH2AFX after 48‐h incubation in range of pHe values tested without BLM treatment. Right panel corresponding to γH2AFX foci/cell at 48 h of pHe incubation. Only foci larger than 10 pixels were counted. Experiments were performed in triplicate. Values are mean ± SEM. *P < 0.05.

The formation of nuclear γH2AFX foci represents a sensitive indicator of DSB repair signaling 24. γH2AFX is required for the accumulation of many DNA damage response (DDR) proteins at DSBs. In normal cells, persistence of repair foci indicates delayed DNA repair kinetics such that residual γH2AFX levels may reflect endogenous genomic instability in tissues and have been associated with precancerous lesions 25. Persistence of γH2AFX foci is also one of the hallmarks of aging 26. As shown above, PFGE analyses indicated that the DSB repair kinetics was reduced by suboptimal pHe when monitored after a 15 min recovery period. We sought to determine whether or not the DSB repair response persists following prolonged recovery periods at suboptimal pHe, this time, by relying on the greater sensitivity of immunofluorescence. Persistence of γH2AFX foci was monitored for given pHe ranging from 7.4 to 6.9 after a recovery period of either 24 h or 48 h (Fig. 2A and Fig. S1). pH stability was carefully monitored throughout the recovery period. After 24 h of recovery from bleomycin treatment, a twofold increase in the number of DSB repair foci per cell was readily observed when the pH of the culture media was lowered to 7.2, while a fourfold increase was induced when recovery proceeded at pH 7.0 (Fig. 2A, right panel). Interestingly, for each suboptimal pHe tested, only a small (≈ 20%) decrease in the DSB repair foci number was observed when the recovery period was extended to 48 h (Fig. 2A, right panel), suggesting stabilization of the repair foci. Because some noncanonical functions have been recently reported for γH2AFX 27, the specificity of the γH2AFX foci for DSB was ascertained by examining the foci size 28 and colocalization with 53BP1 that was confirmed by a strong Pearson correlation coefficient score (Fig. 2A). 53BP1 is an important regulator of the cellular response to DSBs that has been shown to favor nonhomologous end joining (NHEJ) DNA repair pathway over homologous recombination 29. If NHEJ repair pathway are indeed used to a significant extent at suboptimal pHe, DNA ends could be altered at repair junctions 30, raising the possibility that DSB repair in such conditions creates genetic instability. Lowering of the pHe also resulted in an increase in the total levels of γH2AFX as observed from immunoblot analyses, using histone H4 as a control (Fig. 2B). In this case, immunoblot must represent a lesser sensitive assay than immunofluorescence because formation of foci also involves recruitment of preexisting but dispersed nuclear γH2AFX at DSBs sites in addition to the newly phosphorylated H2AFX. Taken together, these observations indicate that suboptimal pHe impacts the DDR repair kinetics leading to the observed persistence of the DSB repair foci in normal fibroblasts. It is also worth noting that without prior induction of DNA damage from bleomycin, incubation of cells at suboptimal pHe did not show formation of repair foci, indicating that the pH itself cannot account for the formation of new repair foci over the 48‐h recovery period under study (Fig. 2C). A pH‐dependent increase in MRE11 detection is also observed in agreement with an upstream signaling for DSB repair 31 (Fig. S1).

**Figure 3:**
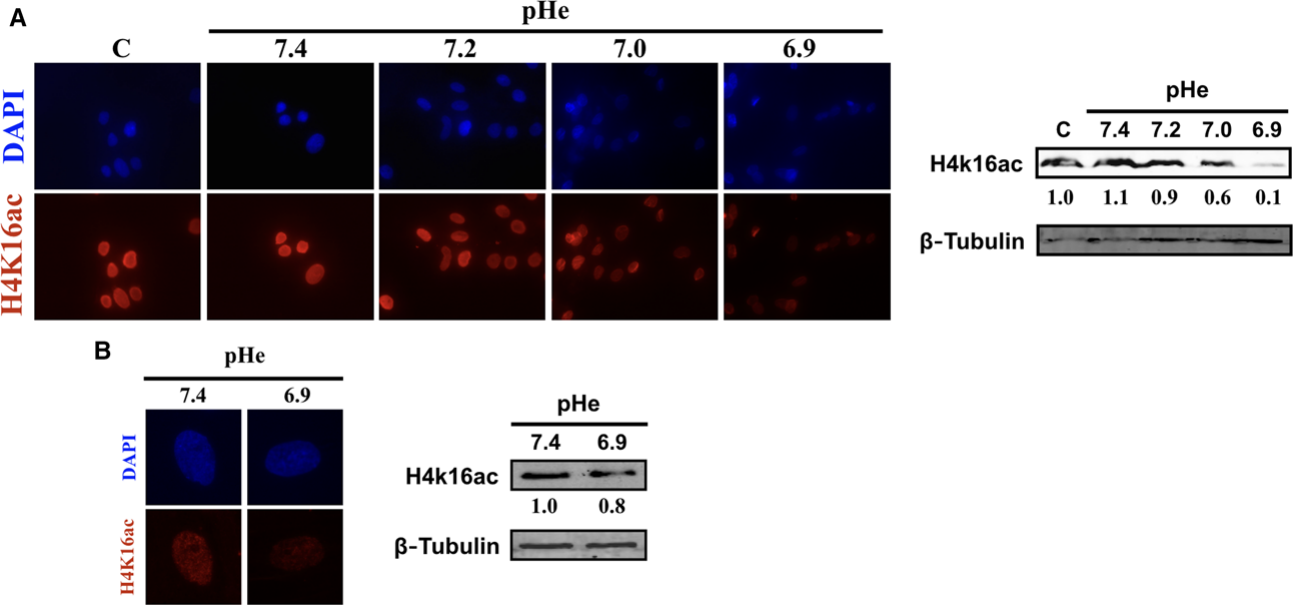
Reduced H4K16ac level at suboptimal pHe. (A) Left panel shows immunofluorescence of H4K16ac in fibroblasts at 48 h postbleomycin treatment. Right panel: immunoblots for the detection of H4K16ac and β‐Tubulin. Quantitation of the H4K16ac signal is shown below for each lane. C, control prior to damage induction. A representative blot of three independent biological replicates is shown. (B) Left panel: immunofluorescence of H4K16ac in fibroblasts at 48 h without BLM treatment. Right panel: immunoblots for the detection of H4K16ac and β‐tubulin. Quantitation of the H4K16ac signal is shown below for each lane. A representative blot of three independent biological replicates is shown.

Decreased acetylation of H4 at lysine 16 (H4K16ac) has been strongly correlated with defective DNA repair 32, 33. It is also a common hallmark of human cancer 34. Using immunofluorescence analysis, levels of H4K16ac were monitored in fibroblasts undergoing recovery following bleomycin treatment, as described above. As shown in Fig. 3A, progressive loss of H4K16ac labeling is observed as soon as the pHe falls under 7.4. The pH‐dependent decrease in H4K16ac was also confirmed by immunoblot (Fig. 3A, right panel). This time, reduction in pH alone, without prior damage, resulted in a reduction in H4K16ac (Fig. 3B). Hence, the pH‐dependent loss of this epigenetic mark correlates with the delayed kinetics of DSB repair and persistence of the DNA repair foci described above. It remains to be established whether the enzymatic pathway for H4K16ac, involving the MOF histone acetyltransferase 35, also plays a role in the process. Interestingly, previous reports showed that catalytically dead human MOF incur a higher frequency of residual DNA DSBs and chromosome aberrations after cellular exposure to ionizing radiation 36, which is in agreement with the reduction in H4K16ac and DSB persistence that is reported here.

**Figure 4:**
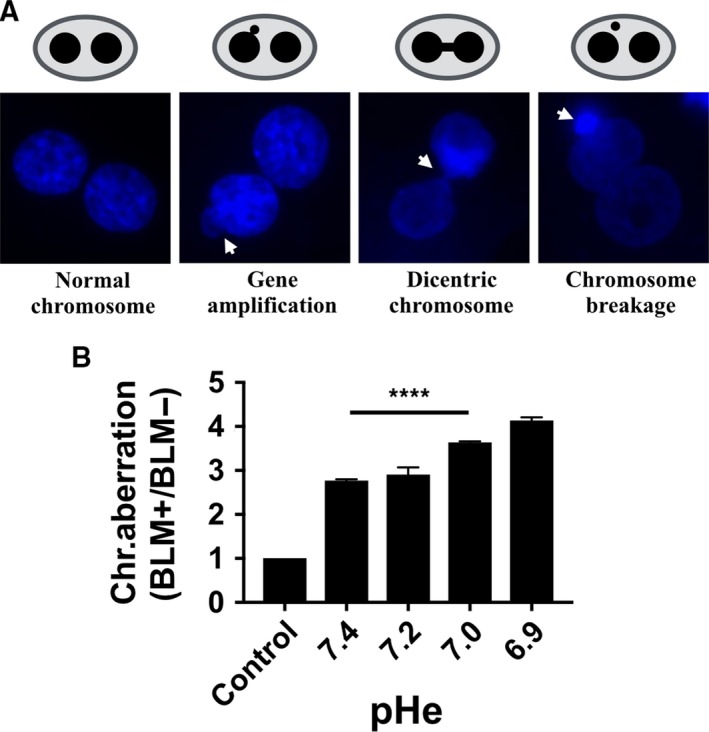
Chromosome instability associated with suboptimal pH. (A) Various chromosome aberrations in fibroblasts as seen by CBMN assay at 48‐h postbleomycin treatment. Images were taken using a Leica DM2500 Optigrid fluorescence microscope and processed with the ImageJ fiji software. (B) CBMN assay in fibroblasts at 48‐h postbleomycin treatment. Control, control prior to damage induction. One hundred cells harboring double nuclei were scored for each given pH. Values are mean ± SEM. *P < 0.05, ****P < 0.00005 from unpaired t‐test.

Bleomycin has been previously demonstrated to induce chromosomal instability in human fibroblasts as detected by micronuclei assays 37. Similarly, we observed that recovery from bleomycin damage at pH 7.4 resulted in a sharp increase in the formation of chromosomal aberrations (Fig. 4A) which are taking various forms including gene amplification, dicentric chromosomes, and chromosome breakage (Fig 4A). Again, departure from the pH 7.4 resulted in a small but significant increase in the residual number of chromosome aberrations that is readily observable from pH 7.2 (Fig 4B). Hence, alteration of DNA repair at suboptimal pH is also reflected at the chromosomal level and suggests a clear impact on genetic stability.

**Figure 5:**
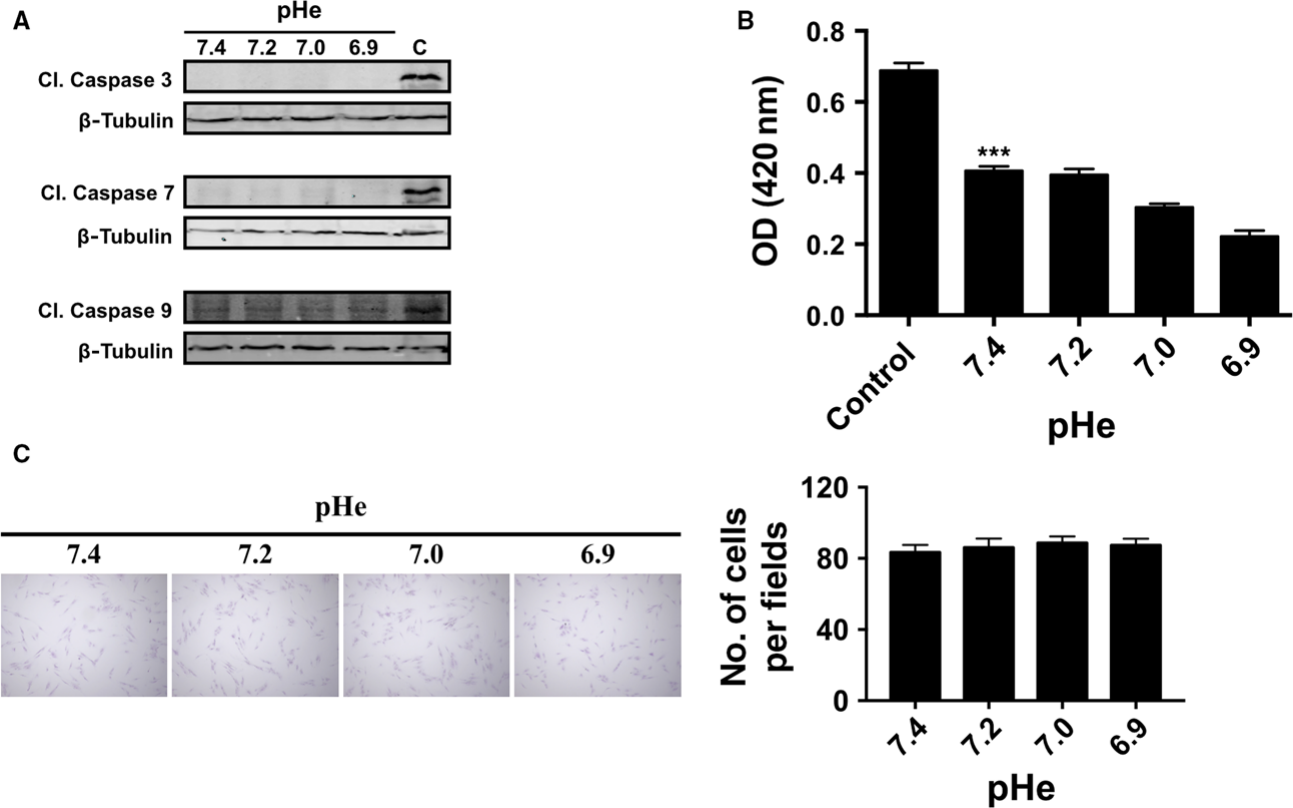
Escape from protective pathways at suboptimal pHe. (A) Caspase cleavage is not activated at suboptimal pHe. Immunoblots of cleaved caspases following bleomycin treatment and 48‐h recovery at given suboptimal pHe. C, Staurosporine (1 μm) was used as a caspase 3 inducer (positive control). (B) Lack of induced senescence at suboptimal pHe. Histogram of β‐galactosidase activity determined by spectrophotometric analysis of ONPG cleavage in fibroblasts following bleomycin treatment and recovery for 48 h at the indicated pH. C, Cells were exposed to 200 μm of H_2_O_2_ as positive control. (C) Recovery from induced damage does not affect cell survival. A crystal violet assay was performed 96 h after BLM treatment. Right panel shows the number of cells/field (*n* = 5) at given pH values. Values are mean ± SEM from three experiments.

Phosphorylation of tyrosine 142 of histone H2AFX (Y142) helps determine whether DNA repair or proapoptotic factors are recruited to chromatin 38. Immunodetection of tyrosine 142 phosphorylation could not be achieved on the fibroblasts used in this study, but whether the pH‐dependent DSBs in response to bleomycin involved apoptosis was tested by monitoring cleavage of caspases 3, 7, and 9 39. As shown in Fig 5A, none of the suboptimal pHe values tested yielded cleavage of the caspases in sharp contrast to the apoptotic inducer staurosporine used as positive control. It is worth noting that caspase‐independent apoptosis may also proceed as a result of increased γH2AFX 40. However, one may probably rule out this possibility because expression of the caspase‐independent mediator AIF 41 remained undetected at suboptimal pHe (not shown). A time‐dependent apoptotic response to bleomycin has been reported when using concentrations of the drug 45 times greater than used in this study 42 or following much longer exposures to a similar concentration of bleomycin 43.

Escape from cellular senescence represents yet another critical and initial event in the transformation of normal cells to tumor cells 44, 45, 46. Activation of the DNA damage response pathways could lead to cellular senescence or apoptosis, and γH2AFX foci have been shown to accumulate in senescing human fibroblasts 47. Although we observed that γH2AFX foci also persist at suboptimal pHe, no evidence of increased senescence could be detected at these pH values based on the spectrophotometric ONPG cleavage test and using H_2_O_2_ as a positive control (Fig. 5B).

We also investigated whether cell recovery at suboptimal pHe may impact survival and proliferation upon prolonged cell culture using the crystal violet assay 23. The same number of cells was treated with bleomycin and allowed to recover at suboptimal pHe, this time, for an extended period of 96 h. As shown in Fig. 5C, none of the suboptimal pHe altered cell proliferation as the number of adherent crystal violet‐stained cells/fields remained unchanged.

**Figure 6:**
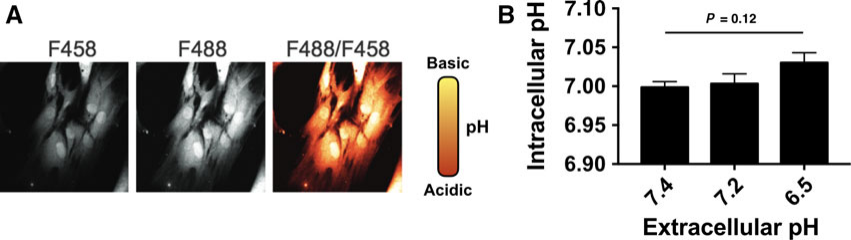
Intracellular pH determination upon fibroblasts' exposure to suboptimal pHe values. (A) Representative fluorescence signal from fibroblasts incubated with BCECF and excited at 450 nm and 488 nm (left and center panels). Picture is shown for a pHe value of 7.4. Spatial map of fibroblast pHi shown as pseudocolored ratio image (right panel). Magnification, 60X. (B) Intracellular pH of 20‐FW cells after incubation at given pHe values (*n* = 3, 30 cells per experiment). Values are mean ± SEM.

Previous studies suggested that modification in pHe induces a concomitant variation in the intracellular pH (pHi) 48, 49, but this may result from specific experimental conditions or cell lines. We sought to establish whether suboptimal pHe induces a concomitant decrease in pHi of the normal human fibroblasts under study. BCECF fluorescence ratio was used as a probe to monitor potential decrease in pHi upon cell exposure to the suboptimal pHe range. As shown in Fig. 6, cells' exposure to suboptimal pHe ranging from 7.4 to 6.5 yielded no detectable change in pHi that is maintained within a narrow neutral range (pH 7.0). Therefore, one may hypothesize that the persistence of unrepaired DSBs and increased chromosomal instability following small pHe deviations is the results of altered pH signaling pathways that may be linked to the DNA repair processes such as the proton‐sensing G protein‐coupled receptors 50.

Taken together, results from these experiments demonstrate for the first time that conditions leading to subtle decreases in pHe, considered as subclinical, may still impact the kinetics of DSB repair, persistence of DSB repair foci, and chromosomal stability in the absence of apoptosis or induced senescence. This state of genetic instability may favor the transition to a precancerous phenotype. We surmise that the deleterious impact of suboptimal pHe would be especially enhanced in patients harboring heritable cancer susceptibility genes encoding DNA repair proteins. In other words, the genetic instability that arises at suboptimal pHe may therefore represent an ‘adjuvant’ carcinogenic factor that would help these poised cells to undergo neoplastic transformation. Strategies aimed at stabilization of pHe would therefore add to the arsenal of cancer prevention strategies.

## Author contributions

JM and GB wrote the manuscript. Experiments were performed by JM, CO and FL. This work was performed under the supervision of GB, JT and CMD.

## Supporting information


**Fig. S1.** (A) Immunofluorescence detection of γH2AFX and 53BP1 following 48 h recovery from bleomycin treatment. Control, sample before damage induction. Colocalization is shown in white; PCC, Pearson Correlation Coefficient score. (B) Immunoblots for the detection of MRE11 and β Tubulin used as loading control. Right panel shows MRE11/βtubulin ratios at the indicated pHe values. Quantification of three independent immunoblots is shown on the right. Values are mean ± SEM. *P < 0.05.
