# Supplementary figures and images for: Suboptimal extracellular pH values alter DNA damage response to induced double‐strand breaks
Source: FEBS Open Bio. 2018 Feb 16;8(3):416–25. doi: 10.1002/2211-5463.12384 (PMC5832969; doi:10.1002/2211-5463.12384)

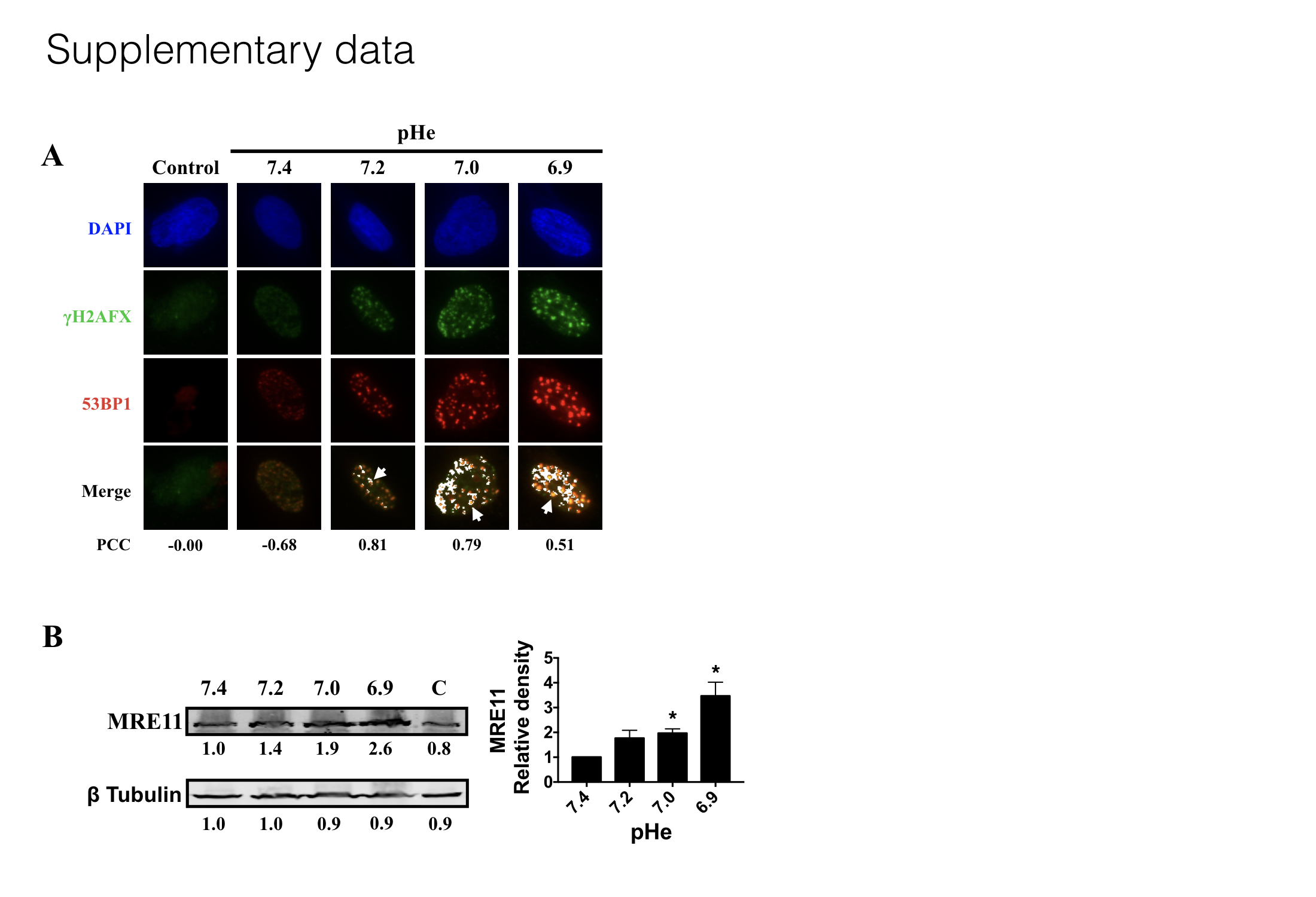

Supplement: Supplementary file 1 — Fig. S1. (A) Immunofluorescence detection of γH2AFX and 53BP1 following 48 h recovery from bleomycin treatment. Control, sample before damage induction. Colocalization is shown in white; PCC, Pearson Correlation Coefficient score. (B) Immunoblots for the detection of MRE11 and β Tubulin used as loading control. Right panel shows MRE11/βtubulin ratios at the indicated pHe values. Quantification of three independent immunoblots is shown on the right. Values are mean ± SEM. *P < 0.05. [file FEB4-8-416-s001.png]
